# Participatory evaluation guides the development and selection of farmers’ preferred rice varieties for salt- and flood-affected coastal deltas of South and Southeast Asia

**DOI:** 10.1016/j.fcr.2017.03.009

**Published:** 2018-05-01

**Authors:** D. Burman, B. Maji, Sudhanshu Singh, Subhasis Mandal, Sukanta K. Sarangi, B.K. Bandyopadhyay, A.R. Bal, D.K. Sharma, S.L. Krishnamurthy, H.N. Singh, A.S. delosReyes, D. Villanueva, T. Paris, U.S. Singh, S.M. Haefele, Abdelbagi M. Ismail

**Affiliations:** a Indian Council of Agricultural Research-Central Soil Salinity Research Institute, Regional Research Station, Canning Town, India; b International Rice Research Institute, Delhi Office, India; c Indian Council of Agricultural Research-Central Soil Salinity Research Institute, Karnal, India; d Govind Ballabh Pant University of Agriculture and Technology, Uttarakhand, India; e International Rice Research Institute, Los Baños, Philippines; f University of Adelaide, Adelaide, Australia

**Keywords:** Farmers’ preference, Participatory varietal selection, Salinity tolerance, Sundarbans, Waterlogging

## Abstract

Rice is the staple food and provides livelihood for smallholder farmers in the coastal delta regions of South and Southeast Asia. However, its productivity is often low because of several abiotic stresses including high soil salinity and waterlogging during the wet (monsoon) season and high soil and water salinity during the dry season. Development and dissemination of suitable rice varieties tolerant of these multiple stresses encountered in coastal zones are of prime importance for increasing and stabilizing rice productivity, however adoption of new varieties has been slow in this region. Here we implemented participatory varietal selection (PVS) processes to identify and understand smallholder farmers’ criteria for selection and adoption of new rice varieties in coastal zones. New breeding lines together with released rice varieties were evaluated in on-station and on-farm trials (researcher-managed) during the wet and dry seasons of 2008–2014 in the Indian Sundarbans region. Significant correlations between preferences of male and female farmers in most trials indicated that both groups have similar criteria for selection of rice varieties. However, farmers’ preference criteria were different from researchers’ criteria. Grain yield was important, but not the sole reason for variety selection by farmers. Several other factors also governed preferences and were strikingly different when compared across wet and dry seasons. For the wet season, farmers preferred tall (140–170 cm), long duration (160–170 d), lodging resistant and high yielding rice varieties because these traits are required in lowlands where water stagnates in the field for about four months (July to October). For the dry season, farmers’ preferences were for high yielding, salt tolerant, early maturing (115–130 d) varieties with long slender grains and good quality for better market value. Pest and disease resistance was important in both seasons but did not rank high. When farmers ranked the two most preferred varieties, the ranking order was sometimes variable between locations and years, but when the top four varieties that consistently ranked high were considered, the variability was low. This indicates that at least 3–4 of the best-performing entries should be considered in succeeding multi-location and multi-year trials, thereby increasing the chances that the most stable varieties are selected. These findings will help improve breeding programs by providing information on critical traits. Selected varieties through PVS are also more likely to be adopted by farmers and will ensure higher and more stable productivity in the salt- and flood-affected coastal deltas of South and Southeast Asia.

## 1 Introduction

Rice is the staple food for about half of the world’s population, and about 90% of the world’s rice is produced and consumed in Asia (Mackill et al., 2012). Rice is a major crop in tropical coastal deltas in South and Southeast Asia. However, several climatic adversities including abiotic stresses like soil and water salinity in both wet (*Kharif*) and dry (*Rabi*) seasons, waterlogging or flash floods, coastal storms and cyclones during the wet season, often affects its productivity (Burman et al., 2013, Islam and Gregorio, 2013, Ismail and Tuong, 2009, Ismail et al., 2010, Singh et al., 2010). Globally, about 230 million ha of coastal areas are saline (Li et al., 2014) of which about 27 million ha are in coastal zones of South and Southeast Asia (Ismail et al., 2010). In India alone, 3.1 million ha are affected by salinity in coastal regions (Yadav et al., 1983).

Rice farming is the primary source of livelihood for millions of poor and smallholder farmers in these areas, despite being sensitive to salt stress, with an upper threshold limit of 3 dS m^−1^ (Maas and Hoffmann, 1977). Above this threshold, rice yield decreases by 12% for every additional unit increase in salinity (ECe, dS m^−1^) (Maas and Grattan, 1999). This sensitivity to salt stress is one of the reasons why the average productivity of rice in coastal delta regions is far below the national average in several countries. For example, in India, the average productivity of rice in this area is less than 1.5 t ha^−1^, which is considerably below the 2.8 t ha^−1^ of the country’s average rice yield (Singh, 2006). High-yielding and stress-tolerant rice varieties are expected to provide a yield increase of about 2 t ha^−1^ in these areas (Ponnamperuma, 1994). However, limited progress has been made in developing and disseminating suitable varieties that can enhance and sustain the productivity of this ecosystem to exploit its considerable potential for food supply.

There is a need to develop stress tolerant varieties adapted to local conditions and also meet the preferences of local farming communities to ensure adoption. Conventionally, breeders evaluate breeding lines and make decisions on release for commercial use, a process that ignores farmers’ preferences. This approach has probably contributed to the slow adoption of new rice varieties, as breeders mostly prefer traits that do not always match the needs of local farmers, such as agronomic and eating quality traits. The participatory varietal selection (PVS; Witcombe et al., 1996) process, where farmers and other stakeholders are involved in the selection of desired breeding lines before their formal commercialization may address this issue to a great extent.

The PVS approach has been successfully implemented in marginal lands before, considering the social context of end users during evaluation, validation, and promotion of new varieties (Joshi et al., 2007, Joshi et al., 2012, Manzanilla et al., 2011, Morris and Bellon, 2004, Ortiz-Ferrara et al., 2007, Paris et al., 2001, Paris et al., 2002, Singh et al., 2010, Singh et al., 2014, Singh et al., 2016). It is designed to include viewpoints of resource-limited farmers, identify varieties suitable for different stress conditions and incorporate a wider range of traits that match specific client preferences (Dorward et al., 2007, Morris and Bellon, 2004, Sperling et al., 2001). Participatory research approaches involving farmers in decision making processes help improve the effectiveness of technology development, increase the speed of adoption and payoffs to agricultural research (Atlin et al., 2002, Ceccarelli and Grando, 2007, Ceccarelli et al., 2000, Freeman, 2001, Gregorio et al., 2004, Joshi et al., 2005, Knox and Lilza, 2004, Tiwari et al., 2009, Trouche et al., 2011, Witcombe et al., 1996).

The Sundarbans represent typical salt-affected areas of the coastal delta regions of South Asia, located between 21° 32′ and 21° 40′ N, and 88° 05′ and 89° 00′ E, in the Ganges delta of eastern coastal part of India. It comprises 102 islands of which 54 are inhabited, spreading across 19 blocks of the two southern most districts of West Bengal, North 24 Parganas (6 blocks) and South 24 Parganas (13 blocks). While the Sundarbans region is one of the richest ecosystems in the world, the inhabitants often face severe poverty, which both contributes to and arises from the vulnerability of the population to a range of natural hazards (World Bank, 2014). The percentage of households below the poverty line in the 19 Sundarbans blocks of South and North 24 Pargans districts is 43.5%, compared with about 23% in the remaining non-Sundarbans blocks of these two districts (CSE, 2012). Farmers in the region are mostly smallholders, resource poor and dominated by scheduled castes and tribes (Mandal et al., 2011). About 90% of the farmers are marginal (<1 ha landholding) and small (1–2 ha landholding). High monsoon rainfall, poor soil, and water quality and natural adversaries like coastal storms and cyclones make agriculture in this region highly non-remunerative, complex and risky.

Rice is grown in about 98% of the cultivated area as a rainfed crop during the wet season. Growing other crops during this season is difficult due to excessive wetting and waterlogging of low-lying fields (Sarangi et al., 2015, Sarangi et al., 2016). In the dry (winter) season, rice is grown on a limited area (20%), and most of the remaining fields are left fallow due to lack of good quality water for irrigation and high soil salinity. Cultivation of rice during the dry season in coastal saline soils is challenging and requires careful choices of suitable rice varieties and good management practices (Sarangi et al., 2014). In both seasons, the productivity of rice is low due to high soil and water salinity, waterlogging, and submergence, besides other problems. Mostly traditional rice varieties are grown during the wet season with little adoption of modern varieties. Evaluation of breeding material in actual field conditions in such diverse ecosystem, and with input from farmers, will ensure selection of proper types and subsequent uptake and adoption.

In this study, we conducted PVS trials on a research station and on-farm locations over seven years in these coastal saline regions. The objectives were to identify stress-tolerant rice varieties suitable for the target areas for use during the wet and dry seasons and to understand smallholder farmers’ preferences and bases of their cultivars’ selection. We also conducted critical analyses to understand the effectiveness of the PVS process, to guide necessary adjustments to ensure selection of appropriate varieties for the spatially and temporally variable production conditions encountered in these regions.

## 2 Materials and methods

### 2.1 Characterization of the experimental sites

**Fig. 1:**
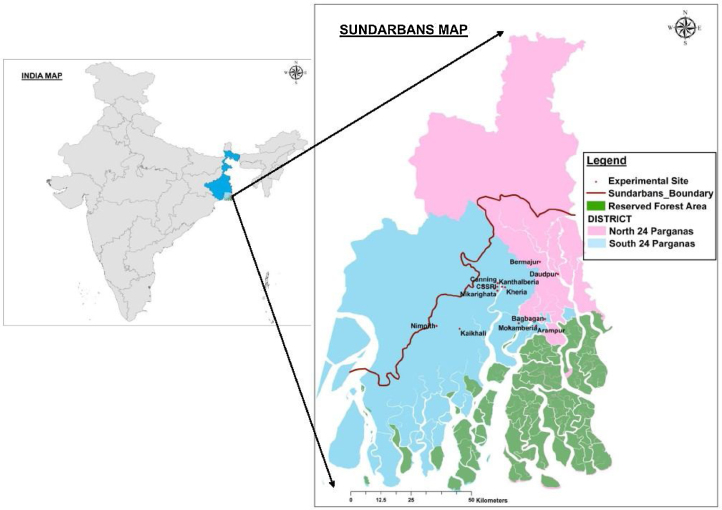
Experimental sites in different locations in North and South 24 Parganas districts in Sundarbans region, West Bengal, India.

The experiments were conducted at the Indian Council of Agricultural Research-Central Soil Salinity Research Institute, Regional Research Station (ICAR-CSSRI-RRS), Canning Town, located in the South 24 Parganas district in the coastal Sundarbans region, and also in numerous farmers’ fields at locations spreading across both South 24 Parganas and North 24 Parganas districts (Fig. 1). Trials were conducted in the wet (*Kharif*) and dry (*Rabi)* seasons during 2008–2014. Soil characteristics were initially determined using standard methods before the start of the experiments. Soil samples were analyzed for organic carbon (Walkley and Black, 1934), available N (Subbiah and Asija, 1956), available P (Olsen et al., 1954), and available K (Hanway and Heidel, 1952). The soil at the experimental sites was mostly heavy textured, varying from silty clay to clay. The top (0–15 cm depth) soil pH ranged from 6.85 to 7.68, and average organic carbon was 0.62%. Available N, P, and K concentrations in the topsoil were 243 kg ha^−1^, 10.5 kg ha^−1^ and 482 kg ha^−1^, respectively.

**Fig. 2:**
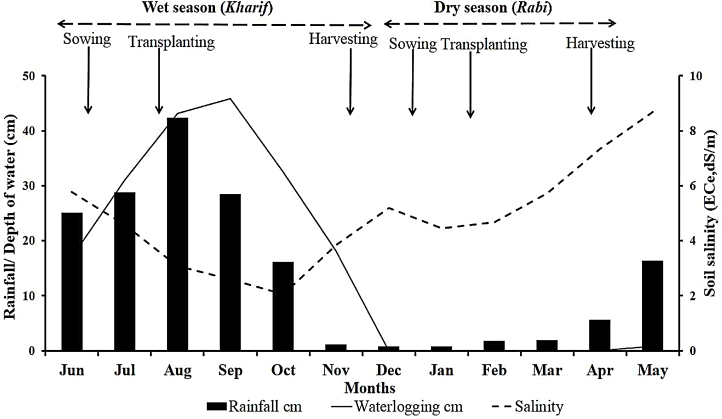
Seasonal variability in soil salinity, depth of floodwater in the field and rainfall during 2008–2014, averaged across sites and months. Arrows indicate the time of different rice cultural operations during the wet and dry seasons.

The climate at the experimental sites is characterized by high monsoon rainfall in a hot and humid summer and a dry, mild winter. Data on weather conditions, hydrology, and salinity were collected from the ICAR-CSSRI-RRS station. The seasonal variability in mean rainfall, surface soil salinity and depth of field water (stagnant water) during 2008–2014, along with the timing of rice cultural operations are presented in Fig. 2. The monthly depth of field water during the wet season and monthly topsoil salinity (0–15 cm depth) were recorded from the experimental fields. Most rainfall is received during May to October. An average of 1630 mm (range of 1140–2160 mm) rainfall was received during the experimental years. Of this, around 85% (range of 80–89%) was during the wet season; and only 6% (1–12%) occurred during the dry season. The mean monthly maximum temperature varied from 24.6 °C in January to 35.4 °C in April and mean monthly minimum temperature was lowest in January (12.9 °C) and highest (26.8 °C) in June. Field waterlogging was deepest in August and September, reaching 40–50 cm at the trials sites. The lowest salinity in the topsoil was about 2 dS m^−1^ reached in October, and the highest was 9 dS m^−1^ in May.

### 2.2 On-station and on-farm participatory varietal selection trials

Promising rice varieties and new breeding lines were evaluated following the PVS approach in on-station and on-farm trials, both managed by researchers (Paris et al., 2011), and in both wet and dry seasons. A total of 17 wet season trials (4 on-station and 13 on-farm) and 16 dry season trials (3 on-station and 13 on-farm) were conducted, each using a randomized complete block design (RCBD) with three replications. The entries included released varieties and new breeding lines recommended from different breeding programs and networks, with 8–13 entries evaluated in each trial. Within a year, the set of entries were the same for all on-station and on-farm trials but were different between years. The entries selected as the best in a preceding year were included in subsequent years along with newly added entries. The entries and the total number of times they have been included in the trials in different years and seasons are presented in Table 1. Plot size varied from 30 m^2^ (5 m x 6 m) to 40 m^2^ (5 m x 8 m), and spacing was 15 cm x 20 cm in all trials. Recommended management practices were used as described in all on-station and on-farm trials.

**Table 1:** Overview of rice genotypes included in various researcher-managed trials in different years during the wet and dry seasons of 2008–2014.

No.	Wet season		Dry season	
	Variety/breeding line	No. of trials	Variety/breeding line	No. of trials
1	Sabita*	17	Lal Minikit (WGL 20471)	13
2	Amal-Mana [CSRC(S) 7-1-4]	17	Khitish	6
3	SR 26B	13	Lalat*	13
4	Geetanjali	15	Satabdi (IET 4786)	13
5	CST 7-1	4	Gontra Bidhan-2 (IET 19571)	13
6	Sumati	2	Boby	13
7	Bhutnath	2	Canning 7	10
8	Utpala	2	CSR 4	7
9	Pankaj	2	CSR 36	3
10	Dinesh	6	CSR 22	6
11	Patnai 23	2	CSR38	3
12	Manasswarabar	5	Annada	13
13	Swarna-Sub1	2	Rasi (IET 1444)	3
14	BINA 8	2	Sankarsaru	3
15	CSRC(S) 21-2-5-B-1-1**	17	Super Minikit	3
16	CSRC(D) 7-0-4**	9	N Sankar	3
17	CSRC(D) 2-17-5**	4	Super Sankar	3
18	CSRC(D) 13-16-9**	4	Parijat	3
19	CSRC(D) 12-8-12**	4	BRRIdhan47	3
20	CN 1039-9**	8	BINA 8	3
21	CN 1233-39-9**	4	IR 64 Saltol**	3
22	CN 12133-3-9**	4	IR 72593-B-18-2-2-2**	3
23	CSRC(S) 47-7-B-B**	1	IR 72593-B-3-2-3-3**	3
24	NC 678	5	IR 76346-B-B-10-1-1-1**	3
25	CR 2006-71-2**	1	IR 76393-2B-7-1-1-3-1**	3
26	CR 2095-181-1**	1		
27	CR 2070-52-2**	1		
28	CR 2094-46-3**	1		
29	IR 76393-28-7**	1		
30	IR 206-29-2-1-1**	1		

*, ** represent ‘check’ and ‘breeding line’, respectively.

Nurseries were established at the start of each season and 40 and 30 days old seedlings were transplanted during the wet and dry seasons, respectively, with 1–2 seedlings per hill. Fertilizers were applied at rates of 120-20-0 and 50-20–10 kg of N-P_2_O_5_-K_2_O ha^−1^ during the wet and dry seasons, respectively. All P and K were incorporated before transplanting, whereas N in the form of urea was applied in three equal splits at seven days after transplanting (DAT), maximum tillering (45 DAT) and at the initiation of flowering (75 DAT) during the wet season. During the dry season, all of P, K and 25% N were applied before land leveling. Half of N was broadcasted 21 DAT, and the remaining 25% was broadcasted at 60 DAT. Hand weeding was accomplished twice (30 and 60 DAT) during both seasons. Chloropyriphos at 2 ml l^−1^ water and tricyclazole at 0.6 g l^−1^ water were used to control insects and diseases, respectively.

### 2.3 Preference analysis

A Preference analysis (PA) through casting votes was conducted during a pre-harvest period when most varieties reached around 80% maturity (Paris et al., 2011). At each PA, a group of male and female farmers and researchers were invited to vote for two most preferred (positive vote) and two least preferred (negative votes) entries, using paper ballots and envelopes placed at the head of each plot. Names of entries were kept anonymous with codes used for each entry throughout the voting process. Votes were then used to identify the most and least preferred rice genotypes and farmers interviewed to understand the reasons behind their choices. This resulted in two types of data; (i) the quantitative preference score (PS) for each variety generated by expressing the number of positive votes minus the negative votes divided by the total number of votes, and (ii) a list of characteristics considered by farmers as the basis for selecting the most and least preferred entries.

### 2.4 Data collection and analysis

Ten hills were randomly selected at harvest from each plot, and plant height was measured from stem base to the tip of the longest leaf or panicle, whichever was longer. The depth of water in the field was monitored using a meter stick installed in each plot. Soil salinity was measured using an electrical conductivity meter in 1:2 (soil: water) and converted to ECe by multiplying with an appropriate conversion factor. At maturity, plants were harvested, then sun dried and weighed to determine grain yield, which was then adjusted to 14% grain moisture content.

The Pearson correlation coefficient (r) was used for correlation analysis between male and female farmers’ PS, between all farmers’ and researchers’ PS and between yield and all farmers’ PS. The ‘r’ value was used for comparing the extent of agreement or disparity in preference choices between any two groups. Growth parameters and yield data were collected from the inner rows, leaving 50 cm border rows at both ends of the plot. Descriptive statistical methods were then used for analyzing the effects of waterlogging and salt stress on rice grain yield.

To rank the various preference criteria, the Rank Based Quotient (RBQ) analysis was employed (Mandal et al., 2013). The criteria used by farmers for their selection of the most preferred lines through PVS trials were listed first, and then they were asked to rank them according to their individual priority on a scale of 1–5. The most preferred criteria were ranked as 1, the least preferred as 5. The analysis allowed ranking farmers’ preferences based on RBQ. A total of 60 farmers (30% females) from 2 locations were interviewed for this preference analysis during the wet season. A similar methodology was used in the dry season. Farmers’ choice of a particular rice variety was influenced by several attributes like salinity tolerance, capacity to withstand waterlogging (plant height), resistance to pests and diseases, grain and straw quality, resistance to lodging and duration of the crop. These attributes were identified from the feedback of farmers during multi-location PVS trials across both seasons and over the years (2008–2014). These attributes were ranked through RBQ analysis during the 2013-14 season. RBQ is a problem identification technique, mathematically presented as follows:

$$RBQ=\Sigma_{j=1}^{n}\frac{fi(n+1-i)*100}{N*n}$$

Where N = total number of farmers, n = total number of ranks (there are five ranks, n = 5), i = the rank for which the RBQ is calculated (for a problem), and f = number of farmers reporting the rank i (for the problem).

Since RBQ scores are index values, tests of significance are not applicable. The data consists of numbers of persons assigning the different ranks, so is considered discrete data, and Chi-square test was used for testing associations between the preference criteria and ranks assigned by the respondents. The null hypothesis “there is no association between preference criteria and ranks assigned by the respondents,” was tested against the alternative hypothesis, i.e. “there exists an association between preference criteria and ranks assigned by the respondents”.

## 3 Results

### 3.1 Preferences of varieties and breeding lines in the wet season

Waterlogging was a major limiting factor for rice growth and productivity at the experimental sites in the monsoon season (Fig. 2 and Table 2). Due to poor or non-existence of drainage facilities, flat topography, low-lying lands and heavy rainfall, the experimental fields remained waterlogged with a depth of more than 30 cm from July to October (Fig. 2). Soil salinity was high (>4.0 dS m^−1^) during early seedling stages (Table 2) when rice is highly sensitive; but lower during reproductive stage, which is also highly sensitive to salt stress. The performance of different rice varieties and breeding lines tested in 17 on-station and on-farm trials during the wet seasons between 2008 and 2013 is summarized in Table 3. The entries were of medium to long duration (140–170 d) and are medium to tall (90–175 cm). Grain yield varied between 2.58–4.39 t ha^−1^.

**Table 2:** The depth of water in the field and top soil salinity (0–15 cm depth) at the two salt sensitive growth stages of rice (early seedling and reproductive stages) during the wet and dry seasons. Values are averages across seasons and years during 2008–2014.

Sensitive growth stages of rice	Wet season (*Kharif*)				Dry season (*Rabi*)	
	Depth of field water (cm)		Soil salinity (ECe, dS m^−1^)		Soil salinity (ECe, dS m^−1^)	
	Range	Mean ± SE	Range	Mean ± SE	Range	Mean± SE
Early seedling	26.0–37.0	31.4 ± 0.60	4.10–5.80	4.58 ± 0.11	3.80–5.60	4.45 ± 0.11
Reproductive	27.0–42.0	33.2 ± 1.00	1.80–2.80	2.05 ± 0.06	4.90–6.90	5.73 ± 0.11

**Table 3:** Agronomic characteristics of rice varieties and breeding lines evaluated in multi-location trials during the wet and dry seasons of 2008–2014. Grain yield represents mean values across the number of trials in which particular entry was used ± SE.

Wet Season				Dry season			
Varieties/breeding lines	Plant height (cm)	Duration (d)	Grain yield ± SE	Varieties/breeding lines	Plant height (cm)	Duration (d)	Grain yield ± SE
CSRC(D) 12-8-12	145–165	165–170	4.39 ± 0.08	Gontra Bidhan-2	105–110	120–125	4.77 ± 0.09
Geetanjali	150–170	165–170	4.12 ± 0.12	BINA 8	90–100	115–120	4.62 ± 0.11
Amal-Mana	150–162	160–170	4.11 ± 0.12	Boby	100–110	125–130	4.34 ± 0.14
Swarna-Sub1	105–110	145–150	4.02 ± 0.33	Annada	95–105	120–125	4.28 ± 0.07
CSRC(D) 7-0-4	140–155	165–170	3.99 ± 0.14	N Sankar	95–105	120–125	4.25 ± 0.06
CSRC(D) 2-17-5	145–155	165–170	3.85 ± 0.06	BRRI dhan47	105–110	120–125	4.25 ± 0.06
CSRC(S) 21-2-5-B-1-1	115–125	140–145	3.84 ± 0.09	IR 76393-2B-7-1-1-3-1	85–95	120–125	4.18 ± 0.23
CSRC(D) 13-16-9	155–165	165–170	3.82 ± 0.07	Lal Minikit	90–105	120–125	4.15 ± 0.15
Manasswarabar	140–145	160–165	3.80 ± 0.32	Parijat	85–95	115–120	4.15 ± 0.04
Sabita	150–165	165–170	3.78 ± 0.14	Super Sankar	90–100	115–120	4.08 ± 0.07
CN 12133-3-9	105–115		3.76 ± 0.10	Rasi	95–105	115–120	4.03 ± 0.08
SR 26B	140–160	165–170	3.71 ± 0.18	Canning 7	95–105	125–130	3.98 ± 0.14
Patnai 23	140–155	160–165	3.63 ± 0.11	Satabdi	85–95	120–125	3.96 ± 0.09
NC 678	155–165	165–170	3.53±0.032	IR 76346-B-B-10-1-1-1	95–1005	120–125	3.92±0.022
BINA 8	90–110	145–150	3.52±0.04	IR 64 Saltol	95–105	120–125	3.91 ± 0.05
CST 7-1	110–120	140–150	3.49 ± 0.13	CSR 4	95–100	125–130	3.82 ± 0.06
CN 1039-9	105–115		3.42 ± 0.02	CSR38	95–100	125–130	3.70 ± 0.12
Sumati	100–105	140–145	3.33 ± 0.17	CSR 22	95–105	125–130	3.65 ± 0.12
CSRC(S) 47-7-B-B	90–100	145–150	3.27	Lalat	100–105	125–130	3.58 ± 0.26
Utpala	105–115	140–145	3.25 ± 0.20	Super Minikit	90–100	115–120	3.54 ± 0.15
CN 1233-39-9	125–135		3.17 ± 0.35	IR 72593-B-3-2-3-3		120–125	3.45 ± 0.23
CR 2094-46-3	150–160	160–165	3.17	Sankarsaru	95–105	110–115	3.36 ± 0.34
CR 2095-181-1	150–160	160–165	3.13	Khitish	85–95	115–120	3.34 ± 0.26
Bhutnath	95–105	130–135	3.13 ± 0.16	IR 72593-B-18-2-2-2		120–125	3.21 ± 0.25
Pankaj	130–150	140–145	3.10 ± 0.14	CSR 36	95–105	125–130	2.19 ± 0.16
IR 206-29-2-1-1	105–115	145–150	3.10				
Dinesh	145–175	165–170	3.09 ± 0.38				
CR 2006-71-2	150–165		2.98				
CR 2070-52-2	145–155	160–165	2.87				
IR 76393-28-7	90–110	145–150	2.58				

The participants in the PA for rice variety selection (681 persons during 2008–2013) were 44% male farmers, 36% female farmers, and 20% researchers. Correlation analysis was performed between the preference scores of male and female farmers, between all farmers and researchers, and all farmers versus grain yield, using Pearson’s correlation (Table 4). Based on farmers’ preferences scores, the four top ranking varieties or breeding lines in each year from the PA are also presented in Table 4. Out of a total of 30 entries, 11 entries were ranked at least once among the top 4, in the 17 on-station and on-farm trials. However the most preferred entries differed across locations and years, and the most preferred entries in a preceding year were not always the most preferred in subsequent years. The most preferred varieties and breeding lines during the six wet seasons (2008–2013) included Geetanjali, Amal-Mana, CSRC(S)21-2-5-1-1, and Sabita. The traits of preference for farmers in the wet season included tall plants, long panicles with more grains, less infestation with pest and diseases, numerous tillers, good grain type, overall good crop performance, enough straw for fodder, thatching and fuel, apparent suitability for parboiling, optimum maturity period, suitability for specific field situations (low/medium land), good lodging resistance, and high grain yield.

**Table 4:** Results of the preference analyses conducted during the wet seasons of 2008–2013.

Year	Trials	Sites	Ranking of most preferred varieties/breeding lines				Correlations between preferences		
			1^st^	2^nd^	3^rd^	4^th^	Male vs. Female	Farmers vs. Researchers	Farmers vs. yield
2008	On-station	Site 1	Amal-Mana	Sabita	SR 26 B	Sumati	0.61**	0.84***	0.35
	On-farm	Site 1	SR 26B	Amal-Mana	CSRC(S) 21-2-5-B-1-1	Sumati	0.75**	0.92***	0.15

2009	On-station	Site 1	CSRC(S) 21-2-5-1-1	Geetanjali	Amal-Mana	Sabita	0.85***	0.90***	0.86***
	On-farm (3)^a^	Site 1	CSRC(S) 21-2-5-1-1	Geetanjali	Amal-Mana	SR 26 B/ Sabita	0.10	0.48	0.96***
		Site 2	CSRC(S) 21-2-5-1-1	Geetanjali	Amal-Mana	SR 26 B	0.62	0.91***	0.19
		Site 3	CSRC(S) 21-2-5-1-1	Geetanjali	Amal-Mana	Sabita	0.85**	0.90***	0.73**

2010	On-station	Site 1	Geetanjali	Amal-Mana	Sabita	CSRC(S) 21-2-5-1-1/ SR 26B	0.91***	0.94***	0.72*
	On-farm (3)	Site 1	Geetanjali	Amal-Mana	CSRC(D) 7-0-4/ CSRC(S) 21-2-5-1-1	–	0.94***	0.91***	0.50
		Site 2	Amal-Mana	Geetanjali	CSRC(S) 21-2-5-1-1/Sabita	–	0.85**	0.81**	0.74*
		Site 3	Geetanjali	Amal-Mana	CSRC(S) 21-2-5-1-1	CSRC(D) 7-0-4	0.93***	0.78*	0.74

2011	On-station	Site 1	CSRC(D) 7-0-4	SR 26 B	Amal-Mana/Sabita	–	0.65^*^	0.48	0.79**
2012	On-farm (3)	Site 1	Sabita	CSRC(S) 21-2-5-1-1	CSRC(D) 7-0-4	CSRC(D) 13-16-9	0.73*	0.71*	0.51
		Site 2	Sabita	CSRC(D) 12-8-12	Amal-Mana	Geetanjali	0.37	0.44	0.67
		Site 3	CSRC(D) 12-8-12	Sabita	Geetanjali	Amal-Mana	0.74*	0.57	0.49
		Site 4	CSRC(D) 12-8-12	Sabita	Amal-Mana	CSRC(D) 13-16-9	0.81*	0.72*	0.88**

2013	On-farm	Site 1	CSRC(S) 21-2-5-1-1	Swarna-Sub1	Patnai-23	Geetanjali	0.81***	0.56*	0.66**
			Sabita	Amal-Mana	Swarna-Sub1	CSRC (S) 21-2-5-1-1	0.75**	0.15	0.87***

*, **, *** significant at *P* < 0.05, 0.01 and 0.001, respectively.

a Numbers in parenthesis in the second column are on-farm trials.

The analyses reflected significant correlation at 5% and 1% levels between preference scores of male and female farmers in 59% of the sites. However, for other sites, a positive but non-significant correlation was observed. Correlations between the preference scores of all farmers versus researchers were strong and significant at 5% and 1% levels in 47% of the trial sites, which is 12% lower than that of male and female farmers. Significant correlations between preference scores of all farmers and yield were observed in only 41% of the sites. This variation indicates more frequent mis-matches between characteristics of highest yielding rice varieties and breeding lines selected by breeders versus farmers’ preferences. Thus, grain yield was not the most preferred trait for farmers in the wet season; other traits seem equally important for the selection of best rice varieties for this region.

Farmers’ preference traits were ranked using the RBQ analysis (Table 5) and the results revealed that the taller genotypes were most preferred, followed by high grain yield. The quality and quantity of straw constituted the third most important trait. Farmers prefer tall varieties (140–170 cm; Table 4) because they are more suited for the lowland situations in coastal areas where land remains waterlogged (>30 cm depth) for about 4 months (July to October). Taller varieties also provide sufficient straw for fodder, for roof thatching and for use as fuel. Resistance to lodging is another important trait preferred by farmers, as they perceive that lodging of tall varieties might cause yield losses.

**Table 5:** Ranking of farmers’ preference criteria for selection of rice entries in the wet and dry seasons. Preference traits were established based on farmers’ feedback during 2008–2013, and ranking was done in 2014 wet and dry seasons, each involving 60 farmers.

Wet season								Dry season						
Preference Criteria	Ranks as assigned by respondents (5 years)					RBQ^a^ Score	Rank	Ranks as assigned by respondents (5 years)					RBQ Score	Rank
	1	2	3	4	5			1	2	3	4	5		
Yield	18	12	10	6	6	62.00	2	24	12	12	10	10	78.00	1
Tolerant to salinity	2	0	2	4	8	10.67	8	22	14	8	6	8	70.00	2
Capacity to withstand waterlogging (height)	10	14	14	14	12	62.67	1	0	0	4	8	8	12.00	8
Pest and disease resistant	4	6	4	10	6	27.33	6	2	8	8	6	4	27.33	5
Quality of straw for thatching/fodder/fuel	10	10	10	8	6	47.33	3	2	2	4	6	8	16.67	7
Resistance to lodging	12	8	8	6	6	44.67	4	0	6	4	8	6	19.33	6
Grain quality for better market price	2	2	4	6	8	16.67	7	2	8	8	6	8	28.67	4
Duration of crop	2	8	8	6	8	28.67	5	8	10	12	10	8	48.00	3
**Chi Square Statistic**						**40.925**							76.082***	

RBQ^a^, Rank Based Quotient.

*** P < 0.001.

Chi-square test was applied to assess associations between the preference criteria and ranks assigned by respondents. For the wet season, the calculated chi-square was 40.925, with *P* value = 0.055. With P > 0.05, we accepted the null hypothesis and concluded that there was no association between preference criteria and ranks assigned by the respondents (Table 5). Hence preference criteria and ranks assigned by the respondents are independent for the wet season. Cyclones with strong winds are common in these coastal areas and can cause considerable damage to standing rice crop. Farmers also selected some genotypes with medium height (100–115 cm) like Sumati, CSRC(S) 21-2-5-B-1-1, and Swarna-Sub1, as in their opinion, they are suitable for medium lands (water depth of 20–30 cm for most of the wet season with no flooding problem). The duration of the crop was another important consideration by farmers. They preferred long duration varieties (160–170 d) like CSRC(D) 12-8-12, Geetanjali, Amal-Mana, CSRC(D) 7-0-4, CSRC(D) 13-16-9, Sabita, SR 26B, and Patnai 23 for lowlands to avoid harvesting in standing water. Medium duration (140–150 d) rice varieties like Sumati, CSRC(S) 21-2-5-B-1-1, and Swarna-Sub1 were targeted for medium-lands. Farmers did not prefer early maturing varieties because they are not suitable for local land conditions and are often damaged by rats and rodents as was evidenced in the trials. Criteria such as pest and disease resistance, grain quality for better market price, and tolerance to salinity ranked least according to the PA. Farmers in this region are mostly small and marginal, growing rice during the wet season for their consumption. They keep some seed for the next wet season planting and sell the only remaining surplus. Farmers preferred bold grain types as they feel that bold types satiate longer after cooking and are also more suitable for parboiling. Because salinity is not a major stress except at early seedling stage, salinity tolerance was not considered important for farmers during the wet season.

### 3.2 Preferences of varieties and breeding lines in the dry season

During the monsoon season, excess salt is mostly washed away with drainage water and pushed down the soil profile. Therefore, salinity stress was lowest in September-October (Fig. 2). However, in the dry season, rice is grown using irrigation, and soil salinity is the major abiotic stress. Salinity built up at the study sites due to gradual drying of the soil after the monsoon season and capillary rise of salinity from the subsoil. Starting in December, topsoil salinity increased progressively and reached a maximum in April-May (Fig. 2). Therefore, soil salinity was high (>4.0 dS m^−1^) during both sensitive growth stages of rice at the experimental sites (Table 2). The plant height, duration and grain yield of different rice varieties and breeding lines evaluated in sixteen on-station and on-farm trials during the dry seasons of 2008–09 to 2013-14 are presented in Table 3. Entries were mostly of short duration (115–130 d), short to medium height (85–110 cm) and with grain yield of 2.19–4.77 t ha^−1^.

To select suitable rice genotypes, 384 farmers participated in the evaluation process from 2008 to 2014. Out of these, 45% were male farmers, 40% were females, and the rest were researchers. A total of 25 entries were included in these trials, 13 of them were favored by farmers and ranked from 1st to 4th (Table 6). The most frequently selected genotypes were Gontra Bidhan-2, Boby, Lal Minikit, and Annada.

**Table 6:** Results of the preference analyses conducted during the dry seasons of 2008–2014.

Year	Trials	Sites	No. of entries	Ranking of most preferred varieties/lines				Correlation between preferences		
				1st	2nd	3rd	4th	Male vs. Female	Farmers vs. Researchers	Farmers vs. yield
2008−09	On-station	Site 1	12	Gontra Bidhan-2	Canning 7	Lal Minikit	Satabdi	0.81**	0.85**	0.86*
	On-farm (2)^a^	Site 1	12	Gontra Bidhan-2	Canning 7	Satabdi	Lalat	0.73**	0.86**	0.69*
		Site 2	12	Gontra Bidhan-2	Canning 7	Satabdi	Khitish	0.93**	0.79**	0.66*

2009−10	On-station	Site 1	8	Gontra Bidhan-2	Annada	Rasi	Boby	0.81**	0.85***	0.86*
	On-farm (2)	Site 1	8	Gontra Bidhan-2	Boby	Rasi	Canning 7	0.93**	0.08	0.72**
		Site 2	8	Canning 7	Annada	Rasi	Boby	0.97**	0.86**	0.16

2011−12	On-station	Site 1	8	Boby	Bidhan-2	Lal Minikit	Annada	0.91*	0.82*	0.89*
	On-farm (3)	Site 1	8	Lal Minikit	Annada	Gontra Bidhan-2	Boby	0.83	0.51	0.66
		Site 2	8	Gontra Bidhan-2	Boby	Annada	Lal Minikit	0.83	0.64	0.70
		Site 3	8	Gontra Bidhan-2	Annada	Lal Minikit	Satabdi	0.93*	0.82	0.73

2012−13	On-farm (3)	Site 1	10	Gontra Bidhan-2	Boby	Annada	Parijat/Super Sankar	0.46	0.39	0.80**
		Site 2	10	Super Sankar	Gontra Bidhan-2	Boby	N Sankar	0.78**	0.52	0.84**
		Site 3	10	Boby	Gontra Bidhan-2	Super Sankar	Annada	0.94**	0.90***	0.32

2013−14	On-farm (3)	Site 1	10	Lal Minikit	Lalat	Satabdi	Boby	0.83**	0.95**	0.65*
		Site 2	10	Lal Minikit	Lalat	Annada/IR 64 Saltol	–	0.20	0.95**	0.68*
		Site 3	10	Lal Minikit	Lalat/IR 64 Saltol	Boby	–	0.83**	0.92**	0.65*

*, ** significant at *P*< 0.05, and 0.01, respectively.

a Numbers in parenthesis in the second column are on-farm trials.

Similar to the wet season, a ranking of the most preferred entries in the dry season varied across locations and years. Preferred entries were selected based on a range of traits includingd tolerance of soil salinity, long panicles with more grains, no or minimum infestation by pest and diseases, more tillers, good grain type, overall good crop performance, sufficient straw for fodder, thatching and for use as fuel, suitability for parboiling, optimum maturity period, and high yield. Correlations between preferences of male and female farmers were significant in 75% of the trials (Table 6). Significant correlations were also observed between preference scores of all farmers versus researchers in 56% of the trials. Similarly, significant correlations between preference scores of all farmers and grain yields were recorded in 69% of all trials, which was much higher than in the wet season (41%). This variation indicates that yield was the most preferred criteria for farmers in the dry season. The RBQ analysis (Table 5) also reflected that ranking of preference criteria was different compared to that of the wet season. The yield was the most critical trait for choosing suitable rice varieties and breeding lines in the dry season. After yield, the tolerance to salinity was 2nd because salinity is a major constraint for farming in coastal saline areas in the dry season. Crop growth duration was another important criterion for selection. Farmers in the region preferred short duration (115–130 d) varieties, because of freshwater scarcity, and together with high soil salinity, most of the land (about 80%) remains fallow during the dry season. Where possible, farmers irrigate their crops from shallow tube wells. Farmers are mostly smallholders with limited resources to invest in purchasing water from neighboring farmers who own shallow tube wells. Soil salinity also builds up gradually as the dry season progresses. Short duration rice varieties will have fewer irrigation requirements and will mature before the build up of high soil salinity towards the end of the season.

High grain quality for better market value was identified as another critical trait for selection. Farmers grow rice in the dry season mostly for marketing because the productivity is higher compared with the wet season. Farmers have better control over crop management, especially water and nutrients. During field days, participating farmers indicated that about 90% of their rice production in the dry season was sold. Consequently, farmers in the study areas preferred rice with long slender grains (Table 4, Table 6). Farmers in general do not keep seeds of the dry season rice varieties for the succeeding dry season because good storage facilities are required to maintain optimum humidity, especially during the hot and humid monsoon months. However, this is less of an issue for storage for the wet season’s rice seed because of the dry conditions after harvest.

Pest and disease resistance and resistance to lodging were also considered important for selection of rice lines for the dry season. Farmers preferred varieties with sturdy culms to avoid lodging and yield losses because of occasional rain and storms that occur during the dry season. Quality of straw and height were ranked as least important compared to other criteria. Farmers preferred medium height (85–100 cm) because they believe it is associated with resistance to lodging.

Chi-square tests were applied to assess the associations between preference criteria and ranks assigned by the respondents. Based on preference ranking under dry season, the calculated Chi-square was 76.082 (*P <* 0.001). This indicated a stronger association between preference criteria and ranks assigned by the respondents in the dry season than during the wet season.

### 3.3 Selection of best varieties based on farmers’ preferences

During the PA, farmers were asked to cast their votes for the two most preferred and two least preferred entries, which allowed ranking of all entries based on cumulative farmers’ preference scores. The ranking of most preferred entries changed from year to year in both seasons (Table 4, Table 6), and even between sites within same years. For example, Amal-Mana, which was included in all 17 wet season trials, had quite different rankings between trial locations and years (Table 4). Similarly, in the dry season, Annada, which was included in 13 out of the 16 trials, had different rankings in different locations and years (Table 6). Based on farmers’ preference scores across locations and years, eight entries in both seasons were ranked 1st or 2nd, while 11 and 13 entries were ranked among the top 4 in the wet and dry seasons, respectively (Table 7).

**Table 7:** Farmers’ most preferred varieties and breeding lines in trials^a^ conducted during the wet and dry seasons of 2008 − 2014, in the coastal regions of the Indian Sundarbans.

Most preferred varieties/lines	Wet season				Most preferred varieties/lines	Dry season			
	Frequency of preferences (no.)^b^					Frequency of preferences (no.)			
	1st rank	2nd rank	3rd rank	4th rank		1st rank	2nd rank	3rd rank	4th rank
Amal-Mana	2	5	7	1	Gontra Bidhan-2	8	3	1	–
SR 26B	1	1	1	2	Boby	2	3	2	4
CSRC(S) 21-2-5-1-1	5	1	3	2	Lal Minikit	4		3	1
Geetanjali	3	5	1	2	Super Sankar	1		1	1
CSRC(D) 7-0-4	1		2	1	Canning 7	1	3		
Sabita	3	3	3	3	Annada	–	4	3	2
CSRC(D) 12-8-12	2	1			Lalat	–	3		1
Swarna-Sub1	–	1	1	–	IR64 Saltol	–	1	1	
Patnai-23	–	–	1	–	Satabdi	–	–	3	2
Sumati	–	–	–	2	Rasi	–	–	3	–
CSRC(D) 13-16-9	–	–	–	2	Khitish	–	–	–	1
					Parijat	–	–	–	1
					N Sankar	–	–	–	1

a Trial details presented in Table 1.

b Frequency of preferences indicates the number of times a particular variety was ranked as 1st to 4th.

## 4 Discussion

Most rural people living in coastal tropical deltas of south and southeast Asia are dependent on agriculture for their livelihood. The majority of them are smallholders, facing severe poverty and regular hunger periods because of low land productivity and limited alternatives. Rice productivity in the coastal region is low and unstable because of the persistence of several abiotic stresses like waterlogging in the wet season and soil and water salinity in both wet and dry seasons (Sarangi et al., 2014, Sarangi et al., 2016). The assumption in this study is that the rice productivity in these areas can be enhanced by developing and disseminating suitable stress tolerant varieties presently not accessible to farmers. Participation of local smallholder farmers in the process of selecting suitable rice varieties is crucial to understand their preferences and to select material adapted to these difficult conditions for subsequent use (Gyawali et al., 2007). The need for participatory approaches to identify such material is highlighted by the fact that conventional breeding has not brought significant crop improvement to smallholder farmers in these marginal environments (Kerr and Kolavalli, 1999, Lipton and Longhurst, 1989, Tiwari et al., 2009). The PVS trial approach is a simple and cost-effective method to understand farmer’s selection process and to identify their most preferred varieties for these less favorable areas. This approach was found successful especially in remote and marginal areas where farmers had limited resources and opportunities (Belay et al., 2005, Joshi and Witcombe, 2002, van Asten et al., 2009, Witcombe et al., 1996). Consequently, germplasm evaluation and selection and knowledge of varietal preferences of farmers were the primary reasons behind this PVS study. Farmers are the key stakeholders for the adoption of new technologies (Antle and Crissman, 1990). The feedback will help rationalize breeding strategies by targeting necessary traits and will also contribute to optimizing resource use (Bhuiyan et al., 2004, Waldman et al., 2014).

Our study showed that characteristics of suitable rice varieties for smallholder farmers in this coastal salt and waterlogging troubled region are variable, as farmers need to adjust to the complex production systems and stresses typical of their local conditions. Preferential traits were obviously different for the wet versus the dry season (Table 5). Sarangi et al. (2016) observed that preference for new varieties by farmers during the wet season largely depends on traits necessary for survival and higher yields in flood-affected areas of the Sundarbans region of West Bengal. In the wet season, uncontrolled waterlogging and poor drainage are the dominant risks that require taller varieties or varieties capable of elongation with rising water (Singh et al., 2011, Kato et al., 2014); therefore, plant height at maturity was the most preferred trait. Because the wet season crop is the main source of food supply for most farmers, grain yield ranked second. The third preference rank is quality and quantity of straw, demonstrating its importance for various uses, including construction of homes, fodder for livestock, and fuel for cooking. The fourth-ranked trait was resistance to lodging, which is of particular importance for taller varieties to maintain their high yield, thus a consequence of the first and second preferences. Least important traits for wet season varieties were market- driven grain quality and salinity tolerance, though the latter might have been ranked higher if the PA was conducted at the sensitive seedling stage.

In the dry season, there is no flooding risk, but salinity increases as the season progresses, and most of the crop is sold rather than used for household consumption. Consequently, farmers prefer high yielding varieties with at least some salinity tolerance, short duration, and good grain quality for high market value. Least important were plant height and straw characteristics. Farmers' overall germplasm choices as well as their preference rankings were, therefore seamlessly rational and emulated the constraints and opportunities of their production environment.

Correlation between preference scores of male and female farmers, an indicative of consensus, reflected a strong to very strong agreement in most of the trials across locations and years, especially in the dry season (Table 4, Table 6). This agreement showed that male and female smallholder farmers in the target areas had similar requirements in new varieties. Women and men often work together for a range of different agricultural operations in the Sundarbans region (Mandal et al., 2011). And because of the common cases of male labor migration in this region, women become the major working force for rice production (Mondal, 2013), reflecting that they have full knowledge of the rice production system and needs. Nevertheless, there remain some discrepancies between male and female preferences. In addition to agronomic characters of new varieties, the PVS process can also be extended to include post-harvest characteristics, where obviously women will have some additional preference criteria such as good eating quality, softness after cooking, and high market value (Chi et al., 2007, Paris et al., 2011).

The relatively low correlation in preference criteria between farmers and researchers during both wet and dry seasons indicated that farmers often had different priorities than the researchers, as also reported before (Singh et al., 2010). Farmers’ preferences and their reasoning are important criteria for breeders to consider when developing new varieties to ensure they will be acceptable by farmers and adapted to local conditions. This concern is particularly highlighted by the weak correlation between the preference scores of all farmers and grain yield, with the latter being a central criterion in most breeding programs. Apparently, farmers consider yield when selecting their most preferred varieties, but also other traits necessary for their local farming conditions (Joshi et al., 1997, Mandal et al., 2002, Manzanilla et al., 2011, Singh et al., 2010, Singh et al., 2014, Singh et al., 2016). Site-specific characteristics other than grain yield are expressly important in the wet season, as reflected by the significant correlations with yield in fewer trials than in the dry season (Tables 4 and 6). The ranking of preference criteria following the RBQ analysis showed that plant height rather than grain yield is the most critical trait for farmers in the wet season, because of the predominance of waterlogging (Table 5).

Another outcome of these PVS trials is that farmers’ preferences are seldom uniform across different locations or years. In this process, farmers select and rank varieties based on visual traits of the standing crop during a field day, usually organized between flowering and maturity (at about 80% maturity in the present study). If for some reasons, any variety does not perform well in a given year or at a specific site, farmers will not select it even if it was ranked high in previous seasons. Misiko (2013) pointed out this problem as a fundamental dilemma for PVS. The farmers who participate in PVS and the final selection mostly see these entries only once during the field day without prior knowledge of their performance throughout the season or in previous years. They also have no information about the environmental conditions at the specific site, e.g., presence/absence of diseases or abiotic stresses like floods or salinity. Farmers might also select entries because of some attractive traits like high yield or profuse tillering, neglecting other important traits. This ‘impulse buying’ (Misiko, 2013) can adversely affect the proper selection of germplasm. Misiko (2013) proposed involvement of participating farmers at different crop development stages to give them a better grasp of the characteristics and performance of the germplasm being evaluated, which then would enable farmers to more accurately select appropriate genotypes for their local conditions. Other studies (Bellon and Reeves, 2002, Paris et al., 2011, Sperling et al., 1993), pointed that selection of rice varieties by smallholder farmers is also influenced by other factors, such as farmers’ socio-economic conditions, available resources, specific needs, and preferences. In-depth discussions with farmers during PA field days at various locations, seasons and years provided similar evidence. Although the exact ranking of entries was not always consistent between sites and years (Table 4, Table 6), several entries were repeatedly among the top four at different sites and in different years. And farmers’ preference criteria (Table 5) did not indicate much ‘impulse buying’ but rather a good understanding of their environment without the necessity for a particular stress occurring at the trial site. Moreover, our results imply that PVS should be conducted over multiple years and locations to be able to judge consistency in farmers’ preferences. Similarly, Thapa et al. (2009) suggested multi-year trials while identifying superior wheat cultivars on resource-poor farms through PVS.

A standard second step in PVS involves further evaluation of one or two top preferred lines in farmer-managed trials in subsequent season(s). However, when only a few top-ranked entries are selected for these trials, there are chances of losing some promising lines early in the process. As was observed in our study, the 2 top ranked entries were not selected as most preferred varieties every year (Table 7). Therefore we would propose that more than two entries (e.g., 4), should be tested in follow-up multi-year researcher and farmer managed trials, to increase the chances of selecting the best and stable varieties that are most preferred by farmers, as some preferences might be site- or condition-specific. Our PVS results indicated that choices of farmers were not always consistent because the preference criteria (also the performance of a particular variety) vary across groups. Increasing the number of most preferred rice varieties while providing more options for the farmers will also contribute to increasing farmers’ acceptance. Additional entries will also increase varietal diversity and provide more stability in these unfavorable areas, as one of the benefits of PVS trial system (Tshewang and Ghimiray, 2010, Subedi et al., 2011, Witcombe et al., 2001).

## 5 Conclusions

The study assessed smallholder farmers’ criteria when selecting new rice varieties suitable for salt-affected coastal delta regions of tropical Asia. The data showed that farmers have different preference criteria for rice varieties for the wet and dry seasons. Farmers’ assessment was multivariate and involves multiple traits, including agronomic characteristics, tolerance of prevailing abiotic stresses, and socio-economic conditions. Farmers have a clear understanding of their rice environment, and the major traits that the new varieties must possess. In addition to grain yield, conventional breeding programs should obviously consider these important and sometimes site and season specific traits such as tolerance to waterlogging, quality and quantity of straw, and lodging resistance for wet season rice varieties. Besides grain yield in the dry season, preferred traits include salinity tolerance, medium to short duration, and good grain quality for better market value. Comparative analyses of the order of ranking of top-preferred entries in these complex ecosystems suggested the need for testing entries over multiple locations and years to select the best performing and stable genotypes. Furthermore, the study suggests preferably more (e.g. four rather than two) better-performing varieties should be retained over the years and promoted through farmers’ managed PVS trials, thereby increasing the chances of the best entries being selected. These findings highlighted the importance and effectiveness of PVS process in these less favorable, marginal, and complex environments in providing useful feedback to inform breeding programs and facilitate the development of adapted varieties that meet farmers’ expectations in coastal regions of tropical South and Southeast Asia.
